# Reaching remote Amazonian communities to eliminate trachoma

**Published:** 2017

**Authors:** Julian Trujillo Trujillo, Tim Jesudason, Girija Sankar

**Affiliations:** Group Coordinator for the Integral Management of Emerging, Re-emerging and Neglected Diseases: Ministry of Health and Social Protection of Colombia.; Communications Specialist: International Coalition for Trachoma Control, London, UK.; Assistant Director of Programs & Communications: International Trachoma Initiative Atlanta, USA.

**Figure:**
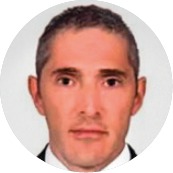
Julian Trujillo Trujillo

**Figure:**
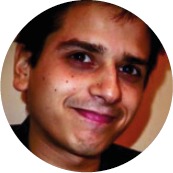
Tim Jesudason

**Figure:**
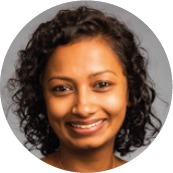
Girija Sankar

**Figure:**
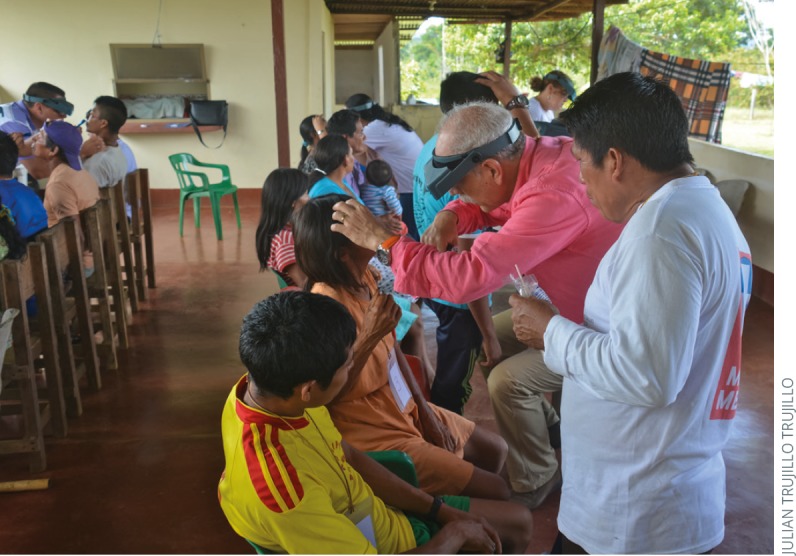
Community members being checked for trachoma in Mitu, Vaupés District. COLOMBIA

Worldwide, many indigenous peoples are at risk of developing trachoma, a bacterial eye disease that disproportionately affects the world's poorest communities. In Colombia's remote Amazonian districts, trachoma is still endemic amongst many of the indigenous communities that live there.

The remoteness of these communities often means they have limited access to formal health care services, which makes trachoma elimination very challenging. However, extraordinary new efforts by Colombian health authorities to tackle trachoma have shown that great progress can be made if there are adequate resources, co-ordination and local engagement.

## Maximising efficiency

From 2012–2016, the Colombian Ministry of Health researched and created maps showing where trachoma was most prevalent in its Amazonian districts. This was needed to identify all people at risk of trachoma, and to understand where resources had to be allocated. Reaching indigenous communities in the Amazonian districts presented major logistical challenges and required substantial resources due to the contrasting landscapes and limited travel routes. Sometimes, flights had to be chartered; at other times, boats had to be carried along trails where rapids or waterfalls interrupted river travel routes.

Once the mapping work was complete and the Government understood the scale of the trachoma burden, the next challenge was to figure out the best way to deliver treatment in these hard-to-reach areas. To maximise efficiency, program managers developed an ‘integrated package’ of interventions. Alongside the distribution of antibiotics for trachoma, health workers also distributed treatments for soil-transmitted helminths. Because under-developed areas are frequently burdened by a number of diseases that thrive in areas with poor access to clean water and sanitation, Colombian health authorities also established intercultural dialogue to educate the communities about the relationship between personal hygiene and good health. This integrated approach delivered treatment and education to over 400 Amazonian communities, significantly improving health in these communities while maximising the impact of resources.

## Tailored programming

Innovative programming approaches were needed in order to deal with the great cultural diversity among the indigenous communities. Community structures, languages, levels of education, migration patterns, environmental conditions and attitudes to health interventions all differed vastly from village to village, and programme staff encountered over 50 different languages throughout the region. Many indigenous communities also live semi-nomadic lifestyles and frequently cross international borders, making prevention, treatment and surveillance programmes difficult to implement and maintain.

In order to tackle these challenges, health workers from the same or nearby districts were recruited and trained, wherever possible. This helped programme staff to gain a better understanding of local cultures and any migration patterns that might affect planned health care programmes. The health workers could also readily translate information into the local language, which increased trust between programme staff and community leaders, thereby increasing community confidence in programme interventions.

Colombian health authorities are also developing working relationships with neighbouring countries and their health authorities in order to overcome the challenges posed by migration. National programme managers meet regularly to discuss trachoma elimination strategies at major events such as the World Health Organization (WHO) Pan-American Health Organization regional meeting, where experiences are shared and relevant courses of action are decided. This regional collaboration has had a positive impact on cross-border interventions and has led to new initiatives, including mapping for trachoma and soil-transmitted helminths in Peru along the Amazonian basin, near the border with Brazil and Colombia.

## Going forward

Despite recent progress, Colombia has more to do to in order to eliminate trachoma as a public health problem by 2020. Around 180,000 people are still at risk of trachoma in remote and hard-to-reach parts of the country. The good news is that Colombia's experience shows that tailoring programmes to fit the needs of indigenous people works. With adequate resources, extensive context-specific planning, extended timeframes and strong consultation with a range of stakeholders, from village chiefs to foreign health departments, programmes can improve health among indigenous communities.

